# How Knowledge of Ancient Egyptian Women Can Influence Today’s Gender Role: Does History Matter in Gender Psychology?

**DOI:** 10.3389/fpsyg.2016.02053

**Published:** 2017-01-05

**Authors:** Radwa Khalil, Ahmed A. Moustafa, Marie Z. Moftah, Ahmed A. Karim

**Affiliations:** ^1^Institute of Pharmacology and Toxicology, Otto von Guericke University MagdeburgMagdeburg, Germany; ^2^School of Social Science and Psychology, Marcs Institute for Brain and Behaviour, Western Sydney UniversitySydney, NSW, Australia; ^3^Department of Zoology, Neuroplasticity and Pain, Faculty of Science, Alexandria UniversityAlexandria, Egypt; ^4^Department of Prevention, Health Psychology and Neurorehabilitation, SRH Mobile UniversityRiedlingen, Germany; ^5^Department of Psychiatry and Psychotherapy, University of TübingenTübingen, Germany

**Keywords:** gender psychology, gender role, discrimination of woman, gender inequality, Ancient Egypt, Arabic countries, social learning theory

## Abstract

A gender role is a set of societal norms dictating what types of behaviors are considered desirable or appropriate for a person based on their sex. However, socially constructed gender roles can lead to equal rights between genders but also to severe disadvantages and discrimination with a remarkable variety between different countries. Based on social indicators and gender statistics, “women in the Arab region are on average more disadvantaged economically, politically, and socially than women in other regions.” According to Banduras’ social learning theory, we argue that profound knowledge of the historical contributions of Ancient Egyptian female pioneers in science, arts, and even in ruling Egypt as Pharaohs can improve today’s gender role in Egypt and Middle Eastern countries. Therefore, this article provides an elaborate review of the gender role of women in Ancient Egypt, outlining their prominence, influence, and admiration in ancient societies, and discusses the possible psychological impact of this knowledge on today’s gender role. We suggest that future empirical research should investigate how enhancing the knowledge of women from Ancient Egypt can improve today’s gender role in Egypt and the Middle East. Bandura’s social learning theory is outlined as a possible framework for future research.

## Introduction

Several studies on gender psychology have revealed that social learning and cultural factors affect gender roles and gender behavior (Cahill, 1986; Hacking, 1999; Francis, 2000; Zosuls et al., 2011). The term “gender role" was first conceived by Money et al. (1955) and describes a set of societal norms dictating what types of behaviors are considered desirable or appropriate for a person based on their actual or perceived sex.

From the early beginning, children gain gender roles from their parents and sociocultural environment. These socially constructed gender roles can lead to equal rights between genders, but also to severe disadvantages and discrimination with a remarkable variety between different countries. For example, in Western countries, such as the USA, women’s average salary is 20% lower than that of their male counterparts, despite working in the same field, having higher education rates, and working longer hours (cf. Bureau of Labor Statistics, 2015). Moreover, Moghadam (2004) showed that “social indicators and gender statistics reveal that women in Arab regions are on average, more disadvantaged economically, politically, and socially compared to regions with similar income levels or at similar stages of economic development, such as Latin America, Southeast Asia, East Asia.” Such gender disparities exist despite the fact that in some Arab countries such as Egypt, there have been huge improvements in the education of women.

In 2011, the United Nations Development Program’s (UNDP’s) Gender Inequality Index rated Egypt 126th out of 148 countries, with an overall value of 0.59, where 1.0 is a perfect score (UNICEF, 2011). These indicators reveal alarming gender-based disparities. The reasons for these disparities are numerous: social norms and attitudes, economic pressures and structural forces help maintain the *status quo*. Remarkably, throughout history, gender roles, especially for women, have been changed.

Here, we argue that profound knowledge of women’s role model especially in Ancient Egypt can improve today’s gender role in Egypt and Middle Eastern countries. According to Bandura’s social learning theory, “individuals are more likely to adopt a modeled behavior if the model is similar to the observer and has an admired status.” Therefore, referring to female Western pioneers such as Marie Curie will certainly not have the same impact on Egyptian schools as referring to models within the same culture. Thus, in this article, we provide an elaborate review of the gender role of women in Egypt from the Ancient to the Coptic period and finally discuss the possible impact of this knowledge on today’s gender role.

## Egyptian Women in Ancient Times

In Ancient Egypt, social dignity was not based on gender, but rather on social status (Jeyawordena, 1986; Robins, 1993; Piccione, 2003; Nardo, 2004; Hunt, 2009; Cooney, 2014). This means that women held many important and influential positions in Ancient Egypt and typically enjoyed many of the legal and economic rights given to the men within their respective social class.

### Social Life

Unlike other ancient societies, women in Ancient Egypt had a high degree of equal opportunity and freedom (Nardo, 2004). Ancient Egyptians (women and men) were firmly equal. Interestingly, ancient sources indicate that women were qualified to sue and obtain contracts incorporating any lawful settlements, such as marriage, separation, property, and jobs (Hunt, 2009). Some of these rights are not given to women in modern-day Egypt. Furthermore, the historian Herodotus witnessed an exceptional display of humanity and equality in Ancient Egypt that was not present in other ancient societies (Tyldesley, 1995).

### Art and Music

**FIGURE 1:**
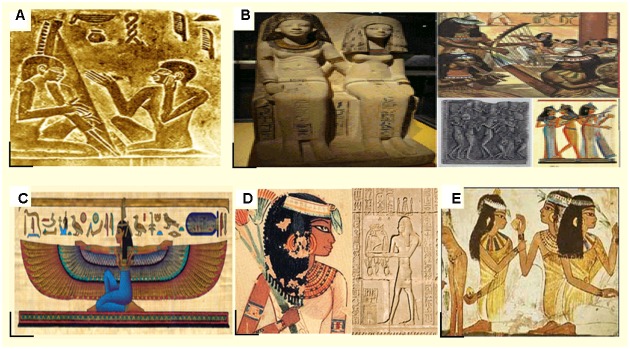
**Examples of remarkable Ancient Egyptian women in art, music, science, and medicine**. Panel **(A)** depicts the harpist **“Hekenu”** and the Cantor **“Iti”** (Source: The Egyptian Museum in Cairo, Egypt). Panel **(B)** shows the statues of **“Nebet”** and her husband from the British Museum in London originally belonging to the tomb of Nebamuncon (1300 B.C.). The Papyri and temple picture on the right side demonstrate the passion of Ancient Egyptian female musicians. Panel **(C)** illustrates the female Goddess **Maat**, who was the symbol of truth during the Ancient Egyptian time. Her energy was supposed to be strongly distributed and spread on the Earth (Source: The original drawing of this papyrus belongs to the temple decoration at Dendera). Panel **(D)** illustrates **Peseshet** on the left side and **Merit-Ptah** on the right side. Both were renowned Ancient Egyptian female physicians. Panel **(E)** is a drawing on the Temple wall of **Tapputi-Belatekallim**, who was an influential scientist and chemist in Ancient Egypt (Source: The British Museum, London, UK).

Gifted women actively participated in the weaving, grieving, and music organizations in Ancient Egypt. Moreover, being a professional in entertainment was another job that was occupied by women in Ancient Egypt (Hunt, 2009).Yet; there is no proof that they ever regulated male laborers, except the most senior imperial women. Hekenu and Iti were two eminent musicians of the Old Kingdom (**Figure 1A**). These women were popular to the point that they even had their performances painted on other people’s tombs, which was a special privilege since it was common to only incorporate individuals from the perished family in the scenes (Hunt, 2009).

Other recorded occupations of Ancient Egyptian women were hair specialists, supervisors of the wig workshop, treasurers, writers, songstress, weavers, dancers, musicians, grievers, priestess’, and even directors of the imperial kingdom. One such recorded occupation was that of “Judge and Vizier to Pharaoh.” This position was held by a woman called “Nebet of the Old kingdom.” The “vizier” was the most capable individual after the lord Pharaoh, going about as his “correct hand man.” However, it is thought that “Nebet’s husband directed in the post whilst she held the title” (**Figure 1B**).

### Marriage

Unlike other societies, Ancient Egyptian women were not subservient to men, were able to choose suitable men for a wedding, and were also able to separate from their husbands (Jacobs, 1996; Hunt, 2009).

Reported contracts affirmed fairness between men and women, expressing women’s rights and ensured equality in employment. For example, an annuity contract found in an archive of Ptolemaic “Family from Suit Nefertiti” illustrates how a divorced Egyptian woman got what was relating to her legacy which she used to share amid marriage. Ancient Egyptian women also had the capacity of unreservedly arranging and fulfilling the terms of her possessions in her contract before going into any marriage plan (Tyldesley, 1994).

Several Papyri indicated the capacity of Ancient Egyptian women to gain wealth independently from their husbands. On the other hand, a husband could lawfully treat his wife as his legal “child,” if he did not want to give any of his riches to his relatives. This way, the wife could acquire the greater part of his riches if they had no children together, or 66% if they had children. The Ancient Egyptian culture believed that a content and delighted home life ought to be the standard. They believed that it could be only accomplished by a husband and his wife, indulgent and administering to one another according to the guideline of Maat (**Figure 1C**) (Tyldesley, 2006).

### Financial Rights

Ancient Egyptian women were able to settle on monetary choices independently, for example when possessing a property. When entering a marriage, women could claim joint property with their husbands. This implies that if the husband was to discard any joint property, then he was lawfully bound to reward his wife with a property of the same worth (Dayan-Herzbrun, 2005).

Inscriptions and sketches portraying women and men going to feasts; hunting and fishing together is an indicator of the equal union of men and women in social life. In addition, tombs enriched with artwork of deceased women dressed extravagantly in the most recent design using aroma, beautifiers, and toiletries. The gifts for life following death were expressions of men’s fondness for their wives.

### Religion

The French Egyptologist, Nobelcouer, indicated the representation of equity between men and women during the Ancient Egyptian times in resembling Gods. For instance, temples, engravings, divider compositions, and also statues displaying powerful and intense female divinities indicates that both genders were regarded as equals and that women were not subservient to men. Prime examples are some prominent female goddesses, including the following: Maat (**Figure 1C**) who had all powers and energy; Isis, the Ancient Egyptian Goddess who is similar to Hathor (Graves-Brown, 2010), the goddess of claiming reverence and recuperating. Most importantly, one papyrus exhibited Isis (Manning, 2012) as in control of certain affairs as much as men. These female divinities were as essential and crucial as male divine creatures for Egyptians during that time. Seemingly, the goddess Bastet, a champion around the vast majority of Egyptian gods, figured out how to enhance women’s well-being, security, and labor. People in Ancient Egypt put Bastet in the same level of respect and admiration as Ancient Egyptian women.

### Medicine

Throughout the ancient history of Egypt, there were more than 100 noteworthy female specialists recorded in every domain of medicine. These women were very educated and highly regarded in their specialization, with pictures showing up on tomb dividers, and hieroglyphics scratched. Among the most imperative female doctors of this time was Peseshet (**Figure 1D**). As seen in engravings found in a tomb of an Old Kingdom, nearly at 3100 B.C.–2100 B.C., she was known as an “administrator of specialists.” Peseshet was a doctor in her specialization, an administrator, and the executive to a group of female doctors. Another significant female doctor from Ancient Egypt was Merit Ptah (**Figure 1E**); she was the first-ever named doctor and the first woman in the historical backdrop of the pharmaceutical field. In this context, she rehearsed pharmaceutical science almost 7,000 years ago, and was deified by her child on her tomb as “The boss doctor.” Another remarkable Ancient Egyptian woman made her print on the field of obstetrics and gynecology, subsequently, in the second century A.D., a doctor named Cleopatra (not the long-dead previous Queen). Cleopatra wrote widely about pregnancy, labor, and women’s well-being, her writings were consulted and examined for more than 1,000 years afterward (Rossiter, 1982).

Furthermore, records exist about Egyptian female doctors, such as Merit Ptah, 2700 B.C and Zipporah, 1500 B.C. Therefore, Egyptian women of ancient times were privileged, as they were able to seek their dreams, strengthen their family life, study, and achieve progress in their work. This led to Egyptian women being among the most regarded doctors of their time.

### Science and Education

Ancient Egyptian women had the right to education. From the age of four, they were trained in instructive establishments, where they were taught science, geometry, and the essentials of hieroglyphic and conversational hieratic. Eventually, they would gain a certificate, the title of ink put holder, and would be authorized for full practice in any of the branches of knowledge they chose. For instance, Egyptian women could attend remedial schools along men or attend a female-only school. One of the greatest examples of the remarkable success of Ancient Egyptian women in science is the one of Tapputi-Belatikallim (**Figure 1E**), who worked with chemicals utilized for aroma generation as a part of Mesopotamia around 1200 B.C.

## Ancient Egyptian Women in Comparison to Ancient Greek Women

Remarkably, women in Ancient Egypt received a larger numbers of opportunities compared to women in Ancient Greece or Rome. For example, Ancient Egyptian women who were educated could study any domain they chose, thus becoming experts in a chosen field (e.g., copyists, researchers, and doctors). Unlike Greek and Roman women who were generally consigned to positions, such as handmaids or housewives, both Ancient Egyptians’ and Greeks’ frameworks of law and social conventions existed side by side in Egypt and operated independently of one another.

From the New Kingdom forward, and surely by the Ptolemaic Period, such proof relates more to the non-first class (i.e., to women of the middle and lower classes). The Greek dominance of Egypt, which started with the triumph of Alexander the Great in 332 B.C., did not clear away Egyptian social and political foundations. By 600 B.C. and the rise of Greek science, the number of women in authentic reports started to expand from 20- to 50-fold every century. This proportion remained moderately consistent for the following 12 centuries.

**FIGURE 2:**
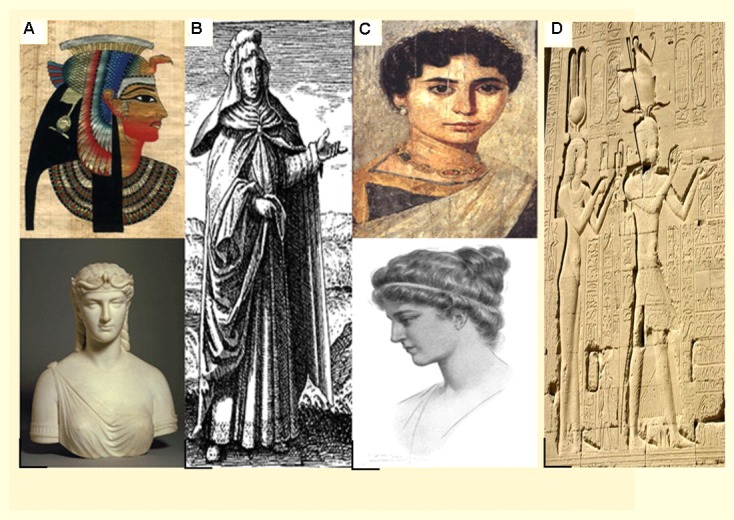
**Remarkable Egyptian women during the Ptolemaic time**. Panel **(A)** depicts **Queen Cleopatra (Cleopatra VII)**. Panel **(B)** illustrates **Maria of Alexandria**, who is also called Maria the Jewess. Panel **(C)** shows **Hypatia of Alexandria** and Panel **(D)** is a photograph of the temple wall of Dendera referring to **Cleopatra VII** and Ptolemy **XV (Caesarion)**.

During the Ptolemaic Period, the preceding colossal human advancements of Ancient Egypt were represented as remarkable. For example, Merit Ptah, who lived circa 2700 B.C.–2500 B.C. is portrayed on her tomb as “The boss doctor;” however, in ancient Greece, which started to be at some point around the eighth century B.C., advancements were principally recorded as male trials. Nevertheless, when the Roman Empire came to its diminishing days in the fourth century A.D., a woman called Hypatia of Alexandria had risen as an image of learning and science. Hypatia, who lived from 370 to 415 A.D., was a mathematician who rose to be a leader of her city’s Neoplatonist theoretical school. Unfortunately, she endured a horrible death because of a Christian swarm, which dishonestly associated her with political interests (**Figure 2C**).

For more than 70 centuries, Ancient Egyptian advancements offered those establishing beginnings, with which mankind guaranteed its human legacy for its incredible fairness for middle-class men and women without any sexism (**Table 1**).

**Table 1:** Table summarizes the names of remarkable women leaders during the Ancient Egyptian time.

Social life	Religion	Education, science, and politics		
Art and music		Medicine	Science	Leading with wisdom
Hekenu Iti	Maat Isis Bastet	Peseshet Merit-Ptah Cleopatra Zipporah	Tapputi-Belatikallim	Queen Heutop
				Queen Khunt
				Queen Abah Hoteb of Thebes
				Queen Hatshepsut
				Queen Ti Queen Nefertiti (Akhnatun’s wife) Cleopatra
				Queen Nefertiti (Akhnatun’s wife) Cleopatra

## Egyptian Women from the Ptolemaic to the Coptic Period

### The Ptolemaic Period

Cleopatra VII gave more careful consideration than her ancestors to the local Egyptian population (**Figures 2A,D**). Although, she was descended from a family of state decision makers, The Ptolemies, who were not Egyptians by blood, but rather Greek/Macedonian (The rulers succeeding Alexander the Great). She was the main Ptolemaic ruler who learned the Egyptian language and adopted all the religious customs to become a genuine Pharaoh. This helped her prominence within individuals outside the Greek rule either in Alexandria (the capital city of Egypt by this time) or in Upper Egypt; where she was worshiped as a divine being (Jacobs, 1996).

Cleopatra supported researchers and exploration at the colossal library of Alexandria, including well-known researchers and mathematicians. Therefore, she appears to have participated with them to the point that the considerable mathematician Photinus called one of his works “Cleopatra’s Canon.” For example, Cleopatra was a gifted linguist and could speak many foreign languages. It was these skills that influenced Julius Caesar’s decision to change the unbalanced counter-intuitive logbook that was used in Rome. This led to the development of the date-book, known as the “Julian timetable” (Feeney, 2007).

### The Coptic Period

In the first or second century, Maria of Alexandria (**Figure 2B**), also known as Maria the Jew, was the first female chemist to concoct the water shower, the three-outfitted still, and other synthetic gear (Reshafim, 2000; Galpaz-Feller, 2004).

The last renowned researcher of the ancient Coptic times was Hypatia (**Figure 2C**), who was born around 360 A.D. in Alexandria, Egypt. She was viewed as the first female mathematician and studied at the University of Alexandria in arithmetic and stargazing. Alongside her father, they developed “Arithmetica,” “Almagest,” and “Components.” Additionally, she was believed to have composed “The Astronomical Canon” in which she created the plane astrolabe, used to measure the positions of stars and planets. Other notable developments include a mechanical assembly for refining water and the hydrometer for measuring the thickness of fluids. She also contributed to theoretical works, book-keeping, and stargazing, and was accredited with the diagramming of the divine (**Table 2**).

**Table 2:** List of most recognized female leaderships during the Ptolemaic time.

Ptolemaic time		
Cleopatra (Cleopatra VII)	Maria of Alexandria (Maria the Jewess)	Hypatia
Queen of Egypt	Chemist	The first female mathematician

However, Egyptian women recently became bound by meeting customarily male criteria for exporting achievement, particularly in science, while being relied upon to fit generally female approaches of individual conduct. The outcome is that numerous young Egyptian women decide to keep a strategic distance from this conflict by evading exploratory professions. Therefore, it is simply not only young Egyptian women who need to change, but more importantly, a watchful evaluation is a necessary requirement for instructing techniques, contracting, and advancing women (Fletcher, 2011).

## Implications for Schooling and Media Psychology

For over seven millennia, women played extraordinary roles in Ancient Egypt. Tragically, in recent times the role and impact of Egyptian women declined dramatically because of many erroneous religious and cultural beliefs. Here, we argue that profound knowledge of female role models, especially in the history of Egypt can improve today’s gender role in Egypt and Middle Eastern countries. According to Bandura’s social learning theory, individuals are more likely to adopt a modeled behavior if the model is similar to the observer and has admired status (Bandura, 1977). Therefore, referring to female Western pioneers in Egyptian schools and in the Arabic media will certainly not have the same impact as referring to models within the same culture. In accordance with social learning theory, Bussey and Bandura (1984) showed that “even children at a lower level of gender conception emulated same-sex models in preference to opposite-sex ones.” Thus, this article provides an important review of female Egyptian pioneers which could be used in schools and in the media to compete with the male-dominated historical role models.

Furthermore, the knowledge of female contributions in Ancient Egypt and historical female pioneers might also improve female self-efficacy since models are important sources of social learning and inspiration (Bandura, 1977). Studies on media psychology provide valuable insights on the large effects of media images, especially on children and youth (Burr, 2001; Karim, 2014). For instance, in a US study, children were asked how often they saw their race on television. Seventy-one percent of White children said they see their race depicted very often, compared to only 42% of African-Americans and 22% of Hispanic-Americans. As for who plays the boss, 71% of all children said someone who is White usually plays the role of boss, while 59% said Blacks typically play the criminal (Burr, 2001). Remarkably, improving the media image of African-Americans since the 1980s already had a remarkable impact on (in-group and out-group) perception of African-Americans compared with the previous decades (Karim, 2014: Karim et al., in press).

Moreover, several studies have shown that gender stereotypes in television and advertising can influence gender-role stereotypes in society, further perpetuating gender roles and gender inequality (Signorielli, 1990; MacKay and Covell, 1997; Oppliger, 2007). For a review on gender inequality in the media in several countries see Matthes et al. (2016).

Accumulated empirical evidence suggest that children and youth can learn a variety of behaviors, such as aggressive acts (e.g., Huesmann and Miller, 1994), letter and number recognition (Rice, 1983), gender behavior and appearance (e.g., Signorielli, 1990; MacKay and Covell, 1997; Lopez et al., 2013), from television and other electronic media devices. However, there are also several debates about the limitations, disadvantages from media learning and the impact of psychological and sociocultural factors affecting how children and youth learn from media representations (see e.g., Schmitt and Anderson, 2002; Collins et al., 2007; Chassiakos et al., 2016; Council on Communications and Media, 2016).

Thus, future studies should empirically evaluate the impact of providing positive female models in school and in the media on the gender role in Egypt and in other Arabic countries and investigate the modulating effects of psychological and sociocultural factors. These findings would have crucial implications for politicians and media campaigns aiming to combat gender inequality and discrimination in these societies.

## Author Contributions

All authors listed, have made substantial, direct and intellectual contribution to the work, and approved it for publication.

## Conflict of Interest Statement

The authors declare that the research was conducted in the absence of any commercial or financial relationships that could be construed as a potential conflict of interest.
